# Effect of spider’s weight on signal transmittance in vertical orb webs

**DOI:** 10.1098/rsos.240986

**Published:** 2024-10-02

**Authors:** Koray Yavuz, A. Mahy Soler, Ramón Zaera, Seymur Jahangirov

**Affiliations:** ^1^ Institute of Materials Science and Nanotechnology (UNAM), Bilkent University, Ankara 06800, Turkey; ^2^ Principia Ingenieros Consultores, Madrid, Spain; ^3^ Department of Continuum Mechanics and Structural Analysis, Universidad Carlos III de Madrid, Madrid, Spain; ^4^ Interdisciplinary Graduate Program in Neuroscience, Bilkent University, Ankara 06800, Turkey

**Keywords:** orb web, sensing, vibration

## Abstract

Spider orb web is a sophisticated structure that needs to fulfil multiple roles, such as trapping prey and transmitting web-borne signals. When building their web, heavier spiders tend to increase the pretension on the web, which seems counterintuitive since a tighter web would decrease the chances of stopping and retaining prey. In this article, we claim that heavier orb-weaving spiders increase tension on the web in order to reduce the attenuation of the vibratory signal coming from the bottom part of the web. We support our claim by first building a detailed spider web model, which is tuned by a tension-adjusting algorithm to fit the experimentally observed profiles. Then, the effects of the spider weight and the web tension on the signal transmittance properties are investigated using state-of-the-art finite element analysis tools.

## 1. Introduction

Spider orb web is a highly efficient prey-capturing net that also acts as a sensing tool for the spider. For the web to be an efficient sensing tool, it should serve two purposes: detecting and locating the prey. In an orb web, radial threads extend from the hub to the frame of the web, which allows the spider to sense signals from any position on the web, so the spider is generally located on the hub (e.g. *Araneus diadematus*) or attaches a signal thread to that point and waits in a retreat (e.g. *Zygiella x-notata*). Spiders use mechanoreceptors called slit sensilla in their exoskeleton to sense movements in the cuticle [1]. Slit sensilla are generally positioned together in a parallel array called lyriform organs, named such because of their similar appearance to a lyre. The majority of these organs are located near the leg joint and have remarkable sensitivity with the sensory threshold between 1.4 and 30 nm depending on the slit in the array, which is used to catch any disturbances on the web [2].

There have been both experimental and computational studies about the signal transmission on spider orb webs. Klärner and Barth showed that spiders could distinguish between airborne and web vibrations and observed predatory behaviour to both transverse and longitudinal sinusoidal vibrations [3]. Masters observed the attenuation characteristics of the signals on the web by creating vibrations on the prey-catching region of the web and measuring them near the hub [4]. Landolfa and Barth observed how the geometry of the web affects the signal transmission [5].

Using finite element analysis, Mortimer *et al*. studied the wave characteristics and showed that the signal amplitude variance sensed by spider legs is sufficient for determining the location and the direction of the source [6]. Otto *et al*. created both a physical and a finite element model of a spider web without the frame and anchors and studied the effect of the spider’s mass and pretension of threads on the frequency response of the web [7]. Morassi *et al*. created a continuous membrane model [8] and studied the eco-localization of the prey impact using both a theoretical model and a finite element model [9].

Previous numerical models provide valuable information on the signal transmission on an idealized web. However, the lack of precise control on the pretension in the web threads prevents comparative studies to understand a spider’s web-building behaviour from being carried out, such as adjusting the tension when building in windy conditions [10] or weight increase [11]. To our knowledge, accurate pretension adjustment of each element on a finite element analysis of an orb web model with anchor, frame, radii and spiral has not been done. Thus, there is a lack of studies on how the weight of the spider and the tension of the web affect signal transmission.

In this study, we present an algorithm to calculate the pretension of the threads to accurately represent a natural spider orb web and the effect of the spider’s weight on signal transmission. Our results show that the spider’s weight changes the tension distribution of the web, which results in poor signal transmittance on certain threads and negatively affects prey eco-localization. Our results suggest that the direct relation between the web pretension and the spider’s weight might be related to correcting these issues.

## 2. Methods and models

**Figure 1.**
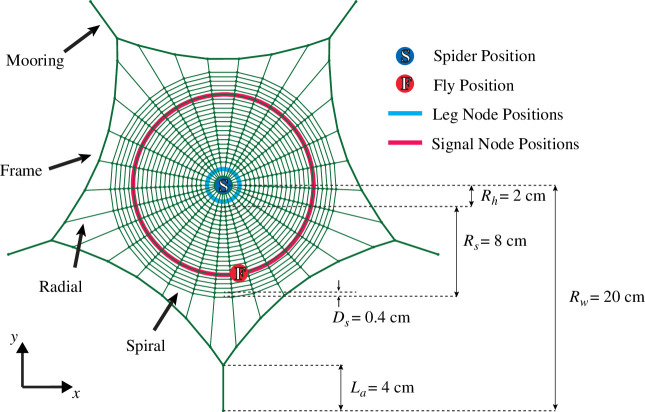
Spider web model used in this study with geometrical parameters. Solid cyan lines show possible positions where the spider can detect incoming signals, which are called leg nodes. A fly can be captured at any point on the solid magenta line, which is called a signal node.

The spider web structure used in this study is modelled after *A. diadematus* orb web, which has four main structural parts: anchor (or mooring), frame, radial threads and sticky spiral (figure 1). Anchor, frame and radial threads have materials with properties different from those of the sticky spiral, the latter being more extensible and less stiff [12], with each having different thickness and pretension values. Using geometric parameters and pretension values found in the literature and implementing the nonlinear mechanical behaviour of spider silk, the model tries to emulate natural webs as closely as possible while using a few simplifications. This model is then subjected to periodic finite deformations representing a captured prey’s struggle in the web. Considering the nonlinearities in the system with finite deformations under a short duration, using a finite element analyser with an explicit integration scheme would be appropriate. For this purpose, simulations were conducted using Abaqus/Explicit 2020.

The following subsections describe how the model is created and the methodology of the simulations.

### 2.1. Geometry of the orb web

The spider web model has five anchor threads connected to five anchor points that act as the rigid support of the whole web, and they are positioned at equal angular distances from each other. The radials also have equal angular distance between them before pretension; however, after the pretension is applied, frames concave inward, and the angular distance between them changes. The spirals were simplified to concentric circles with a constant distance between them.

Natural orb webs generally have a distinct hub architecture [13]; however, neither the tension values of the threads in the hub nor their specific architecture are studied in detail in the literature, so the radii connect in the hub on a single node. The spider’s weight and mass are applied to this node to simulate the effect of a spider sitting on the hub.

### 2.2. Modelling the silk

Spider silk has nonlinear material properties, which can be characterized by the microstructure-based continuum model proposed by De Tomassi *et al*. [14]. This model considers the silk to have two basic parts: a hard and a soft fraction. The soft fraction refers to the amorphous network that shows entropic elastic behaviour, and the hard fraction refers to the ordered network that is rich in stiff crystals, made up of polyalanine sequences arranged in β-sheets, which can form hydrogen bonds. As the silk protein chain is stretched beyond a certain limit, the hydrogen bonds of the hard fraction start to break, transforming into the soft fraction, thus increasing the contour length. Both fractions are included in the calculation of the stress–strain behaviour; the soft fraction is considered to be always active, while the hard fraction consists of active and inactive phases. This study uses a modified version of this model, which was employed in a previous study by Soler & Zaera [15].

### 2.3. Pretension procedure

**Figure 2.**
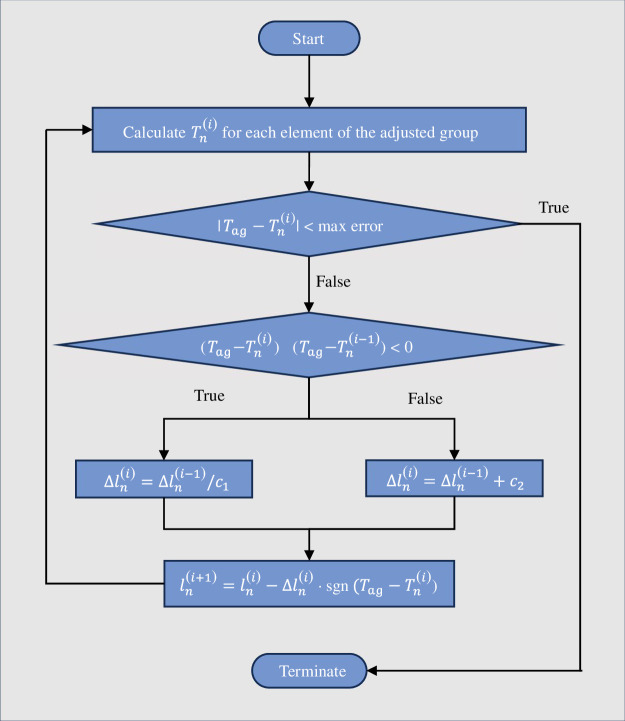
Tension adjustment algorithm flowchart.

Our goal is to compare various realistic tension distributions with respect to spider weight. To achieve this, we developed a method that systematically tunes pretensions of the threads to a desired profile based on experimental studies on *A. diadematus* [16]. Using the material model described in the study by Soler & Zaera [15] and using the diameter value of the different silk types presented by Lin & Sobek [16], a MATLAB algorithm is created to calculate the initial lengths of spiral and radial threads. The algorithm adjusts the length l of each element of the adjusted group in the model to get the desired tension value, $T_{ag}=T_{spi}$ for spirals and $T_{ag}=T_{rad}$ for radii. First, the tension of each element T is found and is compared against the desired tension value $T_{ag}$. If $T<T_{ag}$, the original (unstretched) length of that element is decreased by an amount δl, which increases its tension. For the opposite case of $T>T_{ag}$, the original length is increased by δl. If T oscillates near the target value, δl is divided by a constant $c_{1}>1$ to get closer to $T_{ag}$. Otherwise, δl is increased by a constant $c_{2}$ to speed up the convergence. The algorithm flowchart is shown in figure 2. To apply pretension to the whole web, first, radii are tensed in a model without any spirals. After adding spirals, the same algorithm is applied to them. The frame and anchor lengths are not modified, and the tension value of the anchor and frame threads are the result of the adjustment of the radii and spiral tensions.

The tension values used in this study are $T_{spi}=10$ μN and $T_{rad}=100$ μN. The resulting average pretension values after the calculated thread lengths are implemented in Abaqus can be seen in table 1. The radials have different tension values at different parts due to the tension of spirals, and the radial tension is higher than average close to the frame and lower close to the hub due to the concavity of spiral threads toward the web centre. The average radii tension close to the frame is 122 μN, and the average spiral tension value is 9.7 μN, which are compatible with the values presented by Lin & Sobek, 132 μN and 10 μN, respectively [16].

**Table 1.** Pretension values and diameter of the threads in the model.

type of thread	diameter (μm)	average pretension (μN)	standard deviation
spiral	2.4	9.7	0.11
radial	3.93	102.5	15.7
frame	7.23	962.5	62.0
anchor	8.03	1741	0.66

### 2.4. Modelling the prey

The prey is considered to be a fruit fly stuck to the centre node of the sticky spiral thread that has a distance of 8 cm from the hub. Possible positions of the fly are called signal nodes. The average weight of the fruit fly is 10 μN (BNID 110030 [17]), which is simulated as a constant downward force at the ‘signal node’ that corresponds to the position where it got stuck. In order to simulate a prey struggling on the web, the signal node is moved in a sinusoidal pattern with 1 mm amplitude at 200 Hz, which is used by Wignall *et al*. in real spider webs to create predatory behaviour [18] and is close to the wingbeat frequency of *Drosophila melanogaster* [19]. The signal node is moved in three ways: for transverse waves, the node is moved perpendicular to the web plane; for longitudinal waves, it is moved in-plane toward the hub; and for lateral waves, the movement is parallel to the spiral it is connected to. The spider is thought to be able to sense the excitations on the radii from points close to the hub, which are referred to as leg nodes. These excitations can be the tension changes on the web silk or the displacement of part of the silk that is under tension. The possible positions of signal nodes and leg nodes can be seen in figure 1.

## 3. Effect of spider’s inertia and weight on signal transmission

**Figure 3.**
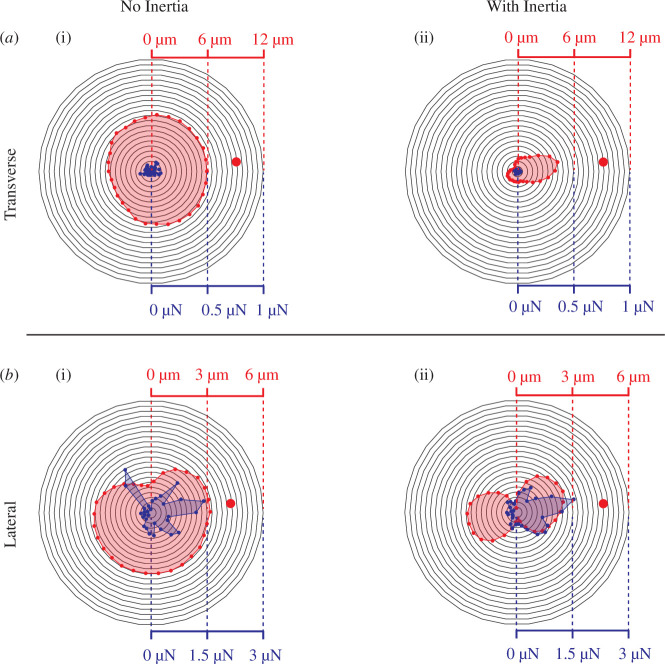
Displacements and tension change values near the hub, shown by the red and blue colours, respectively, caused by the signal source located at the red dot. Web’s response to transverse excitation is shown at (*a*) and to lateral excitation at (*b*). (i) Spider web with 490 μN downward force applied to the hub but no inertia and (ii) 50 mg spider at the hub. For transverse waves, tension changes on the radii are tens of nanonewtons at maximum and do not show any significant change for different cases. The displacement is affected by the addition of inertia, and positional information for the signal location becomes significantly better compared to the no inertia case. Similar observations are present for the tension change values due to the lateral waves.

The presence of the spider at the centre of the web affects the mechanical response of the web in two different ways. On the one hand, the spider’s inertia modifies the system’s dynamics and, thus, the transmission of waves that help the spider recognize the direction of the signal source [9]. On the other hand, the spider’s weight exerts a load that alters the distribution of pretensions on the web, which also translates into a modification of its dynamic response, affecting the transmission of the dynamic signal. We wanted to decouple the effect of inertia and the load to understand the effect of each, since for some of the orb weaver spiders such as *Z. x-notata*, the spider is not present at the centre, but it attaches a signalling thread to the hub which it keeps under tension. This behaviour presents a case where only a load is present at the centre of the web. The simulations from Mortimer *et al*. showed that this signalling thread does not contain any directional information [6], which is consistent with the observation that once the spider senses a motion from the signal thread, it first runs to the hub and only then does it head toward the source of the vibration [3]. In our simulations for transverse waves, we observe a minimal difference in the displacement of radials near the hub when no inertia is present at the hub, even when its weight is present as a downward force at the hub (figure 3*a*). When the spider’s inertia (along with its weight) is included, some of the energy created by the vibration is spent accelerating the spider’s inertia. This obstructs the vibratory signal, and it is not transmitted past the hub. Thus, directional information becomes much more precise. This is consistent with the result presented by Zaera *et al*. [9], but in their model, no weight force was applied. Our result shows that this effect is observable even in vertical orb webs when the spider’s weight is applied. Similar precision betterment by inertia can be seen for other wave types, lateral (figure 3*b*) and longitudinal, albeit to a lesser extent. Results for the longitudinal waves are presented in the electronic supplementary material.

## 4. Effect of spider’s presence on the hub on signal transmission

Tension on the web and signal transmission are related. On a single strand of silk, larger tension lowers the signal attenuation [20]. This phenomenon seems to be used by spiders. An orb weaver from the family Uloboridae, *Octonoba sybotides*, increases the tension on the web when hungry, which allows it to sense smaller prey [21]. *Cyclosa octotuberculata* pulls the radii while waiting for prey, which increases its chances of sensing and catching the prey [22].

Heavier spiders build webs with higher tension [11]. This is a counterintuitive response since higher tension on the threads would diminish the spider’s chances of stopping and retaining prey [10,23]. Since the majority of the orb weavers sit at the hub, upper radii would have even higher tensions compared to a lighter spider web. We hypothesize that this behaviour might be related to the spider’s sensing abilities. As the spider sits on the hub, the tension on the bottom radials would be smaller, and on the top, it would increase. Spiders generally put more importance on the bottom part of the web; the majority of vertical orb web-building spiders sit face downward on the hubs of their webs. As the spider gets heavier, it builds webs with more vertical asymmetry where the area of the bottom part is larger than the top part [24,25]. This is thought to be a result of the spider’s ability to run faster downward compared to upward, and vertical asymmetry is a way of optimization for catching prey as it can reach every part of the web at similar time periods [26]. The bottom part is also different structurally: there are more radials at the bottom, and the spiral mesh is denser [27]. Material investment can also be different, which is the case for *A. diadematus* webs. Ecribellate orb spiders such as *A. diadematus* cover their spirals with a sticky aqueous solution that acts as a glue to ensnare prey caught on the web [28]. This aqueous glue coating quickly forms droplets, which are significantly larger at the bottom part [29], thus increasing the chances of capturing prey. These features point to the bottom part of the web being more valuable for catching prey. Therefore, the spider needs to be able to distinguish signals coming from the whole web but specifically from the bottom part.

**Figure 4.**
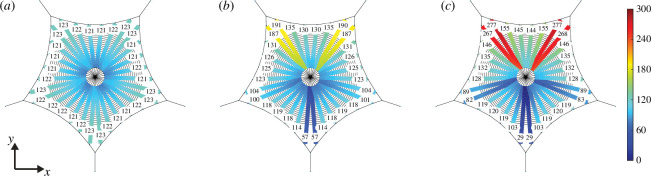
Prestresses in the web with $T_{rad}=100$ μN: (*a*) no spider present at the hub, (*b*) 490 μN and (*c*) 980 μN weight applied at the web centre, representing two different spider weights in *y* direction. The numbers present the pretension values of radii close to the frame. All the values shown, including the colour bar, have units of μN.

In a vertical orb web, the weight of the spider creates a tension variance on the web, with tension values decreasing below the hub and increasing above (figure 4). If the mass of the spider is higher than 75 mg, the tension of the bottom radii gets so small that the segments close to the hub start to sag and signal transmission to the hub is eliminated completely.

**Figure 5.**
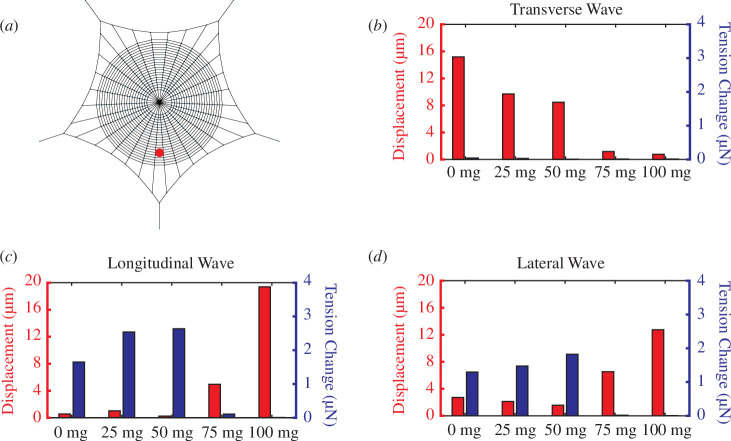
Average displacement and tension change of leg nodes on two radii closest to the signal location is shown with changing spider mass for $T_{rad}=100$ μN. The signal source is located at the bottom region, shown by the red dot on the spider web model (*a*), and the wave types of the vibration are transverse, longitudinal and lateral for (*b–d*), respectively. For transverse waves, the displacement decreases as the mass of the spider increases. For lateral and longitudinal cases, tension change is not observable for 75 and 100 mg cases, and the increase in displacement comes from the silk that sags and is not under any tension.

Decreasing tension on the radii negatively affects signal transmission, which can be seen by the responses generated by all three wave types (figure 5). This may adversely impact prey capture, especially for a small prey that would generate lower amplitude signals.

**Figure 6.**
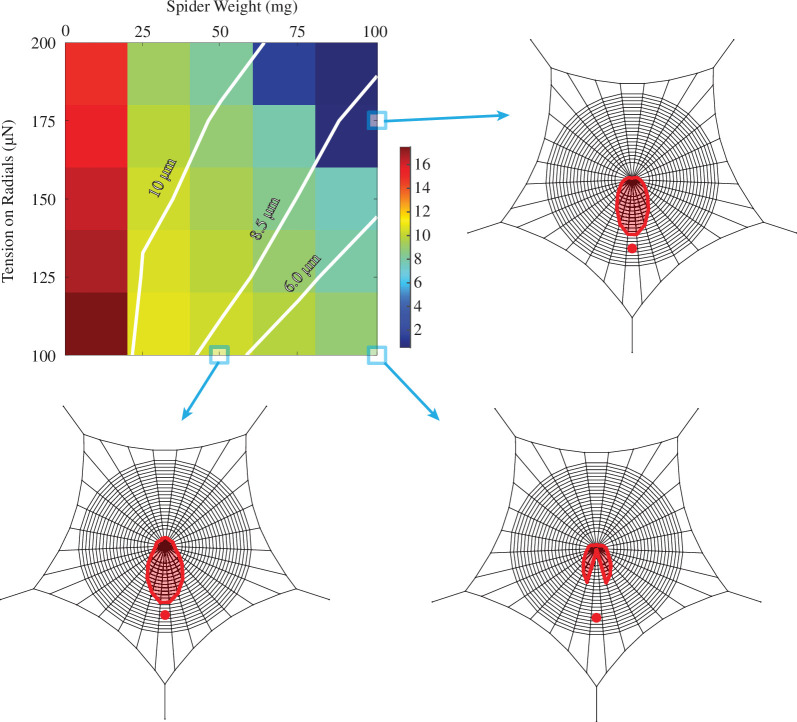
Contour map of signal amplitude with respect to the spider weight and radii pretension ($T_{rad}$) for the vibration generated at the bottom part of the web, with white lines showing the equivalue points. Three points on the map are shown as web models. The signal source is shown as the red dot, and the displacements on the radii are shown with red solid lines, where a larger distance from the hub represents a larger value on the radial.

**Figure 7.**
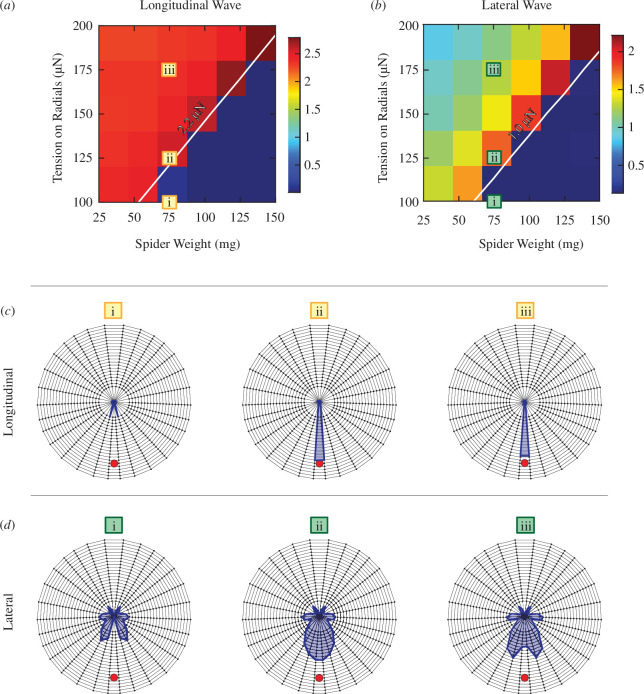
Contour maps for (*a*) longitudinal and (*b*) lateral waves. The signal amplitude is represented as tension change on radii, with colour bar representing the values with units of μN, plotted with respect to spider weight and radii pretension ($T_{rad}$). Model representations corresponding to the areas marked on the contour map are shown in (*c*) for longitudinal and (*d*) for lateral waves. The signal source is shown as the red dot, and the tension change on the radii is shown with blue solid lines, with a greater distance from the hub representing a larger amplitude. The signal amplitude created by the longitudinal waves does not seem to change much with changing tension (*a*); however, when the tension on the radii becomes too low, the signal is completely lost. Lateral waves also lose signal transmission below a certain (radii tension)/(spider weight) ratio threshold as seen in (*b*). Unlike the longitudinal excitation, for the lateral excitation case, higher tension of the radii negatively affects signal transmission, and the locational information becomes less precise above a certain value.

In order to confirm the previous statement, contour maps of signal transmission amplitudes for different spider masses and radial pretensions are shown in figure 6 for transverse waves and in figure 7 for longitudinal and lateral waves. One can see that increasing the radial tension indeed recovers the previous signal amplitudes near the hub. When a part of the web is loose, locational information is lost, which can be seen in the web models presented in figures 6 and 7. If the signal is not transmitted properly from every part of the web, the excitations coming from the other radials might direct the spider onto the wrong sectors of the web, or the signal can be too weak to detect.

All the wave types are affected by the signal transmission problems caused by silk with very low tension; however, their responses to higher tension differ. Transverse waves are positively affected by the higher web tension, and lateral waves are negatively affected. Out of the three wave types, longitudinal waves are the least affected by the tension change.

## 5. Conclusion

Using a web model with accurate mechanical nonlinear silk properties and controllable pretension, we tried to explain the seemingly contradictory behaviour of vertical orb web weavers building higher tension webs as their weight increases. Our results support our hypothesis that this tension increase is required for the web to transmit vibrations created by captured prey and for the spider to locate its position correctly. We show that when the radial thread tension is low and the spider’s weight is high, the web can generate confusing responses by eliminating signal transmission from certain parts of the web. The direct relationship between the signal amplitude and radii tension might also explain the observations made for spiders such as *C. octotuberculata*, which tenses the radii to increase its chances of detecting prey [22]. Another takeout from this study can be the crucial effect of the spider’s inertia assisting in finding the direction of the signal source, which might explain the behaviour of spiders such as *Z. x-notata* first arriving at the hub after sensing a vibratory signal through their signal thread, instead of choosing the shortest path [30,31].

This study highlights the effect of spider’s weight on the tension distribution of the web, which in turn affects the spider’s web-building behaviour. Spider’s web is considered a sensory organ of the spider in addition to being a trap for prey capture, and spider’s own body seems to be linked to its functionality, as discussed in the previous sections. Our spider web model can be further improved for more realistic results. Different hub architectures can also be included, which might have different properties for improving signal transmittance; however, experimental studies on this particular part are lacking. Another structural simplification in our model is the lack of asymmetry that is naturally present in vertical orb webs, where the bottom part would be larger as the spider gets heavier. The effect of weight on the tension distribution of the web is most likely to be more pronounced in asymmetric webs due to the larger bottom sector with more radii compared to the top. There are various modifications spiders make when a parameter such as the weight is changed due to the many roles that the web is required to fulfil. Singling out a particular aspect, such as tension distribution in this study, is generally needed to be able to understand a spider’s web-building behaviour.

## Data Availability

All data used in this paper are extracted from the literature and cited. The data and code necessary to reproduce the analysis presented in the paper are available from the Zenodo repository [32,33]. Electronic supplementary material is available online [34].
